# Assessing the Causal Effect of Circulating Protein‐To‐Protein Ratio on the Risk of Morbidity of Hepatocellular Carcinoma

**DOI:** 10.1002/cam4.70570

**Published:** 2025-01-07

**Authors:** Qiuyuan Yue, Xiaoxia Li, Xiaoye Wan, Xi Lin, Yueming Li, Mingwei Zhang, Shaohua Xu

**Affiliations:** ^1^ Department of Radiology, Clinical Oncology School of Fujian Medical University Fujian Cancer Hospital Fuzhou Fujian China; ^2^ Department of Radiology The First Affiliated Hospital of Fujian Medical University Fuzhou Fujian China; ^3^ Department of Radiotherapy, Cancer Center The First Affiliated Hospital of Fujian Medical University Fuzhou Fujian China; ^4^ Key Laboratory of Radiation Biology of Fujian Higher Education Institutions The First Affiliated Hospital of Fujian Medical University Fuzhou Fujian China; ^5^ Department of Blood Transfusion FuZhou Second General Hospital Fuzhou Fujian China; ^6^ Department of Hepatobiliary and Pancreatic Surgery, Clinical Oncology School of Fujian Medical University Fujian Cancer Hospital Fuzhou Fujian China

**Keywords:** circulating proteins, hepatocellular carcinoma, Mendelian randomization, protein‐to‐protein ratio

## Abstract

**Objective:**

Several observational studies have identified an association between plasma proteins and hepatocellular carcinoma (HCC). This study aimed to explore the potential causal relationship between the circulating protein‐to‐protein ratio and the morbidity risk of HCC.

**Methods:**

Genetic association data for circulating plasma proteins and 2821 protein‐to‐protein ratios were sourced from the UKB PPP and Suhre's study. Genetic association data for HCC were sourced from the FinnGen cohort (finngen‐R11‐HCC) and the IEU OpenGWAS project (ieu‐b‐4953). Subsequently, a two‐sample Mendelian randomization (MR) and drug‐targeted MR approach were used to evaluate causality associations. To bolster the robustness of our findings, we conducted a series of sensitivity analyses.

**Results:**

Eight protein–protein pairs were identified as causal factors for HCC in the two independent cohorts. For each standard deviation increase in protein–protein pair expression, susceptibility to HCC fluctuated from 0.4974 (95% confidence interval [CI]: 0.2506–0.9871) for the LAT2/SPRY2 protein pair to 1.9763 (95% CI: 1.3009–3.0026) for the ERBIN/LAT2 protein pair. However, among the significant protein pairs, only one circulating protein, TDRKH (odds ratio: 0.5964, 95% CI: 0.4196–0.8476), was causally associated with HCC.

**Conclusion:**

Using multiple datasets and methods, eight protein–protein pairs were identified as having causal associations with HCC. Protein–protein interactions can provide meaningful findings beyond simple pQTL analysis.

## 1 Introduction

Hepatocellular carcinoma (HCC) is one of the most common malignancies globally. The morbidity and mortality rates of HCC are particularly high in certain regions [1] and among patients with chronic hepatitis, including hepatitis B and C [2]. Early detection of HCC is challenging because of its typically asymptomatic and rapidly progressing nature [3]. Primary treatments for HCC include surgical resection, liver transplantation, radiotherapy, chemotherapy, and targeted therapy; however, their effectiveness in enhancing patient survival rates is limited [2]. Among these treatment options, liver transplantation is considered the best option for patients with cirrhosis combined with HCC because of its ability to completely remove the lesions and replace the functionally impaired liver [4]. However, the shortage of liver donors and the high cost limit its widespread application. Moreover, the high recurrence rate of HCC remains a major challenge in current treatments, severely affecting patients' long‐term survival [5, 6]. The mechanisms underlying HCC morbidity are complex and varied. Recent research has shed more light on the roles of genetic mutations, abnormal signaling pathways, and the tumor microenvironment in the development of HCC [7]. Despite advances in targeted drug therapy, its efficacy still falls below expected levels [8], indicating that exploring new therapeutic targets is crucial for treating HCC.

Research has indicated that circulating proteins (pQTLs) are potential biomarkers for assessing HCC risk [9]. Proteins such as alpha‐fetoprotein (AFP) and des‐γ‐carboxythrombotic acid precursor have been used for clinically diagnosing and monitoring HCC. However, their effectiveness is limited by low sensitivity and specificity [10]. Additionally, circulating tumor DNA (ctDNA), as an emerging biomarker, has garnered significant attention in cancer research in recent years. ctDNA refers to DNA fragments released by tumor cells into the bloodstream, and its presence and changes can reflect the genomic characteristics of the tumor [11]. Because of its advantages of being noninvasive, allowing for real‐time monitoring, and possessing high specificity, ctDNA shows great potential in the early diagnosis of cancer [12], predicting recurrence [13], and guiding treatment [14, 15]. However, despite its significant advantages in cancer research, the detection of ctDNA still faces challenges in terms of sensitivity and specificity. In contrast, proteomics, as a method for studying protein expression and function, offers another potential source of biomarkers. Combining ctDNA with proteomics can complement the strengths of both, providing more comprehensive and accurate tumor information [16]. This multilevel biomarker analysis is expected to offer clinicians more precise diagnostic and therapeutic guidance, thereby improving patient prognosis. Recent studies on the role of circulating proteins and their ratios in predicting the risk of HCC have revealed complex interactions between various biomarkers and disease progression [17]. Furthermore, the boundaries between diagnostic serum biomarkers and therapeutic targets are becoming increasingly blurred, and circulating proteins, such as AFP, GPC3, and SALL4, have become biomarker‐guided precision medicines for HCC [18]. However, these studies were observational or cross‐sectional studies, and the existence of confounding factors cannot reveal the causal relationship between these single circulating proteins and their biological links with HCC, leading to low‐quality research evidence.

Mendelian randomization (MR) is a powerful alternative that uses genetic variation as an instrumental variable to infer a causal relationship between exposure and outcomes, thus providing a higher level of evidence [19, 20]. Traditional statistical methods used to study the relationship between biomarkers and disease risk are often affected by confounding factors and reverse causality, making it challenging to establish causal relationships. The MR method randomly assigns genes from parents to offspring, mimicking the randomization process of a controlled trial and minimizing confounding effects. Additionally, research on cell genomics suggests that studies based on a single protein, ignoring the relationships between proteins, lead to false negatives and reduce the level of evidence [21]. To address these deficiencies, this study presents the first protein–protein pair‐based (rQTL) MR study, which highlights the causal association between the biological link between proteins and the pathogenesis of HCC compared with pQTL‐based MR. To date, no MR study has assessed the causal association between the circulating protein‐to‐protein ratio and HCC.

This study used large HCC cohorts and multi‐omics data to explore the causal relationship between circulating protein‐to‐protein ratio and the risk of HCC through MR from the perspective of pQTL and rQTL, with the aim of elucidating the molecular mechanism of HCC. We believe that our findings will identify new insights that may serve as biomarkers for early detection and risk classification and provide new directions for targeted therapy of HCC.

## 2 Methods

**FIGURE 1:**
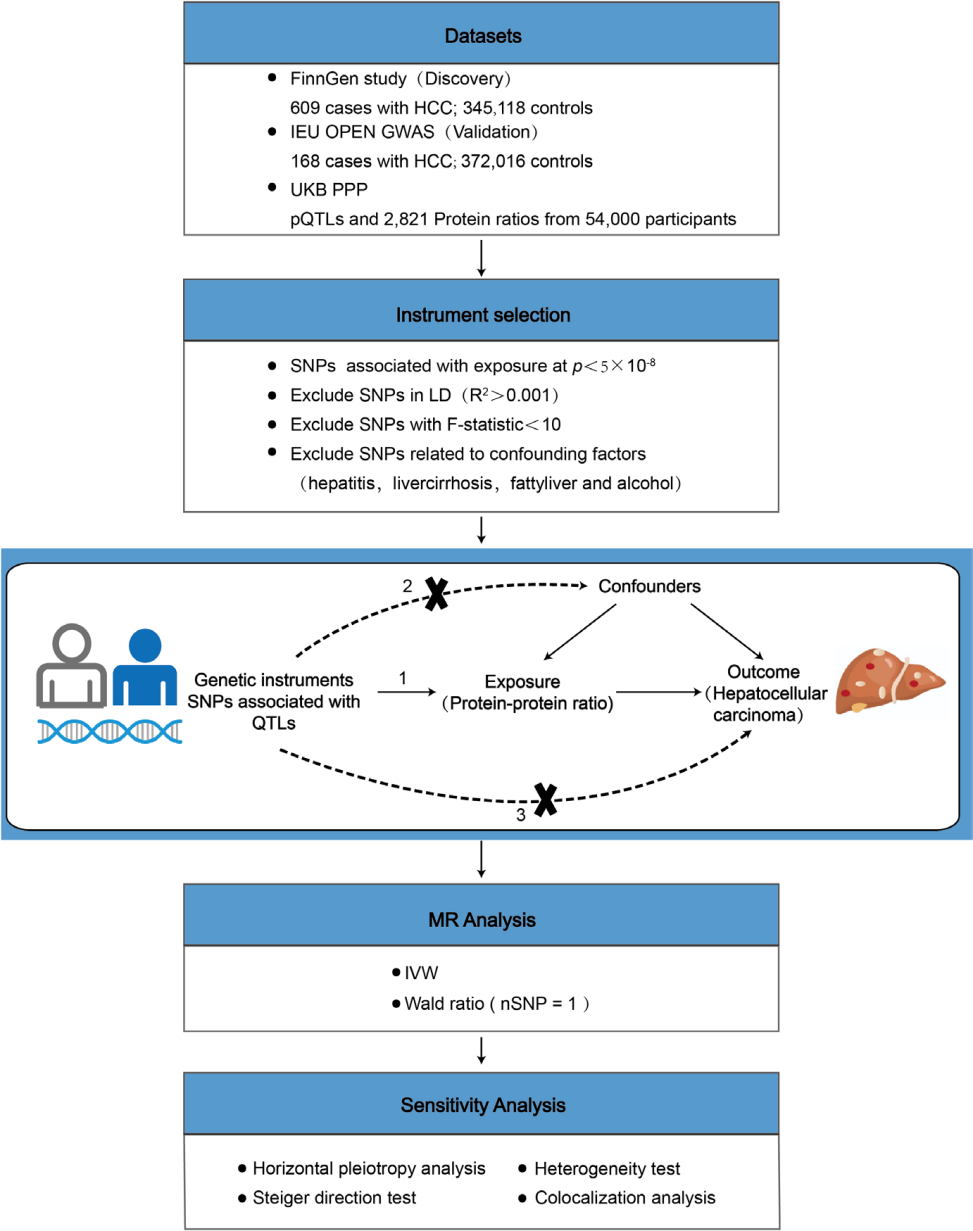
Study workflow of the Mendelian randomization (MR) analysis.

In this study, we explored the potential causal relationship between the circulating protein‐to‐protein ratio and morbidity risk of HCC using two‐sample MR analysis, and strengthened the validation of the causal association using the Steiger directionality test and colocalization analysis. We further analyzed the causal association between the expression abundance of individual proteins in positive protein–protein ratios and the morbidity risk of HCC. The study design is illustrated in Figure 1.

### 2.1 Data Acquisition

Genetic association data for protein–protein ratios were sourced from Karsten Suhre's identification of 2821 protein‐to‐protein ratio‐associated single nucleotide polymorphisms (SNPs) (rQTL) [21]. Genetic association data for circulating plasma proteins (pQTLs) were obtained from the UKB PPP reported by Suhre [22]. Genetic association data for HCC were sourced from the FinnGen cohort (finngen‐R11‐HCC, with 609 patients and 345,118 controls; https://www.finngen.fi/en/) [23] and the IEU OpenGWAS project (ieu‐b‐4953, with 168 patients and 372,016 controls; https://gwas.mrcieu.ac.uk/datasets/) (Table 1).

**TABLE 1:** QTLs and GWAS database information in MR studies.

Type	Data source	Sample size	Cases	Population
rQTLs	UKB‐PPP	54,000	—	European
pQTLs	UKB‐PPP	54,000	—	European
HCC	FinnGen	345,727	609	European
HCC	IEU OPEN GWAS	372,184	168	European

### 2.2 Endpoint Definition

#### 2.2.1 finngen‐R11‐HCC

The endpoint is delineated by a set of registry filters that include the ICD‐10 code C22.0 for the cause of death, ICD‐O‐3 topography code C22, a range of morphology codes from 81700 to 81705, and a behavior code of 3, which together specify the criteria for identifying HCC cases within the registry. Controls for the endpoint excluded individuals who were defined as C3_CANCER or C3_CANCER_WIDE. (https://r11.risteys.finregistry.fi/endpoints/C3_HEPATOCELLU_CARC_EXALLC#).

#### 2.2.2 ieu‐b‐4953

Liver cell carcinoma cases were defined using the following parameters: first, individuals with a site‐specific cancer code (ICD10: C22.0 and ICD9: 1550). Second, site‐specific cancer morphology (behavior) was dealt with using the following rules: Cancer behaviors including “Malignant, primary site,” “Malignant, microinvasive,” “Malignant, metastatic site,” and “Malignant, uncertain whether primary or metastatic site” were included in the dataset. Cancer behaviors including “Benign,” “Uncertain whether benign or malignant,” and “Carcinoma in situ” were excluded from the dataset. Third, individuals with an ICD10: D code but no C code were not included as cases. Controls were defined using the following parameters: individuals who do not have any cancer code (ICD9 & ICD10—C and D codes). And individuals who have no self‐report of cancer [24].

### 2.3 Instrumental Variable Screening

The selection criteria for instrumental variables for plasma rQTLs in conventional two‐sample MR analysis [25] were as follows [26]: (1) *p* < 5 × 10^−8^ was selected as the selection criterion. The coefficient of linkage disequilibrium *R* ^2^ was set as 0.001 [27], the width of the linkage disequilibrium region was 10,000 kb, and the minor allele frequency (MAF) was > 0.01 to ensure the independence of each SNP and remove the influence of linkage disequilibrium on the results; (2) the variables showed no pleiotropy; (3) the instrumental variables were independent of the confounders. Additionally, instrumental variables with an *F*‐statistic > 10 were preferentially chosen to reduce weak instrumental variable bias [28].

The screening criteria for instrumental variables of plasma pQTLs in the MR analysis of drug‐target were as follows: (1) the screening criterion was *p* < 1 × 10^−5^, the linkage disequilibrium coefficient *R* ^2^ was 0.3, the linkage disequilibrium region width was 100 kb, and the MAF > 0.01. This was to ensure the independence of SNPs and mitigate the influence of linkage disequilibrium on the results; (2) the variables showed no pleiotropy; (3) each instrumental variable was independent of confounding factors; (4) the instrumental variable was located in the *cis*‐acting region of the drug target gene (± 1 Mb).

The screened relevant SNPs were extracted from genome‐wide association studies (GWAS) summary data of the outcome variable, HCC.

### 2.4 Co‐Localization Analysis

Co‐localization analysis of positive protein‐to‐protein ratio and individual proteins with HCC was performed using the “coloc” package of R [29]. In a given region, co‐localization analysis assumes that each of the two traits has at most one true causal variation in the region, resulting in five mutually exclusive model hypotheses (H0–H4). The five models assume all possible associations under the following assumptions: H0: phenotype 1 (GWAS) and phenotype 2 (QTL or GWAS) are not significantly associated with all SNP sites in a genomic region; H1/H2: phenotypes 1 or 2 are significantly associated with SNP sites in a genomic region; H3: phenotypes 1 and 2 are significantly associated with SNP sites in a genomic region, but are driven by different causal variable sites; and H4: phenotypes 1 and 2 are significantly associated with SNP sites in a genomic region and are driven by the same causal variable site. The colocalization regions for pQTL–GWAS and rQTL–GWAS were set at ±500 kb from the location of the top‐associated SNP. Strong co‐localization was observed when PH4 > 0.8. Moderate co‐localization was observed when 0.5 < PH4 < 0.8. Weak co‐localization was observed when 0.25 < PH4 < 0.5 [30].

### 2.5 Steiger Direction Test

Steiger's direction test was performed to verify the directionality of the causal association between the eight positive protein–protein pairs and HCC. The variance explained by the instrumental variable SNPs for the protein‐to‐protein ratio and the variance of HCC were calculated using Steiger's directional test [31], which tests whether the variance of HCC is smaller than that of the protein‐to‐protein ratio. If the variance of the outcome is less than the variance of the protein‐to‐protein ratio, it is judged as “TRUE,” indicating that the causal relationship is consistent with the expected direction, while a “FALSE” result indicates that the causal relationship is the opposite of the expected direction.

### 2.6 Statistical Analysis

Statistical analyses were conducted using R version 4.4.1 (R Foundation for Statistical Computing, Vienna, Austria; www.r‐project.org). The “TwoSampleMR” package, version 0.6.6, was employed for general two‐sample MR and drug–target MR analyses. The Wald ratio method and inverse‐variance weighted approach [32] were used to assess the causal associations between HCC, the protein‐to‐protein ratio, and protein abundance using QTLs as instrumental variables. The intercept of the MR‐Egger method was used for pleiotropy analysis. SNP heterogeneity was assessed using Cochran's *Q* test [28]. *I* ^2^ was also used to represent the proportion of total variation due to heterogeneity. *p* < 0.05 was considered statistically significant.

## 3 Results

### 3.1 Causal Association Between Circulating Protein‐To‐Protein Ratio and HCC

#### 3.1.1 Two‐Sample MR

**FIGURE 2:**
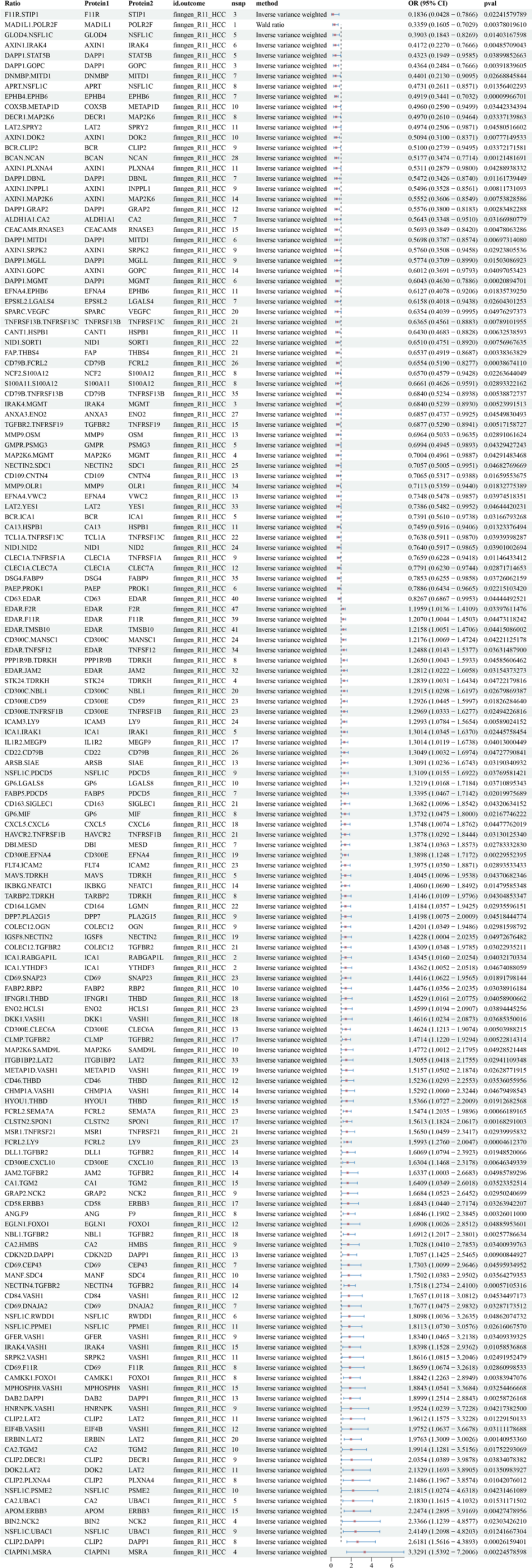
Primary analysis of causal associations between protein–protein ratio and HCC pathogenesis using a FinnGen database.

This study identified 2821 protein‐to‐protein ratios. In the FinnGen database, 40,028 SNPs associated with HCC were screened for MR analysis after excluding confounders such as hepatitis, cirrhosis, fatty liver, and alcohol consumption (Table S1). The screening results are detailed in Data S1. The main results of the analysis of the causal association between the protein‐to‐protein ratio and HCC morbidity risk are shown in Figure 2. The results revealed that LAT2/SPRY2 (odds ratio [OR]: 0.4974, 95% confidence interval [CI]: 0.2506–0.9871; *p* = 0.045), FAP/THBS4 (OR: 0.6537, 95% CI: 0.4919–0.8687), ICA1/IRAK1 (OR: 1.3014, 95% CI: 1.0345–1.637; *p* = 0.024), ERBIN/LAT2 (OR: 1.9763, 95% CI: 1.3009–3.0026; *p* < 0.005), and ITGB1BP2/LAT2 (OR: 1.5055, 95% CI: 1.0418–2.1755; *p* = 0.029), a total of 93 protein pairs, were associated with increased risk of HCC. The results of the heterogeneity and horizontal pleiotropy analyses of rQTL associated with HCC (finngen‐R11‐HCC) are shown in Data S2.

**FIGURE 3:**
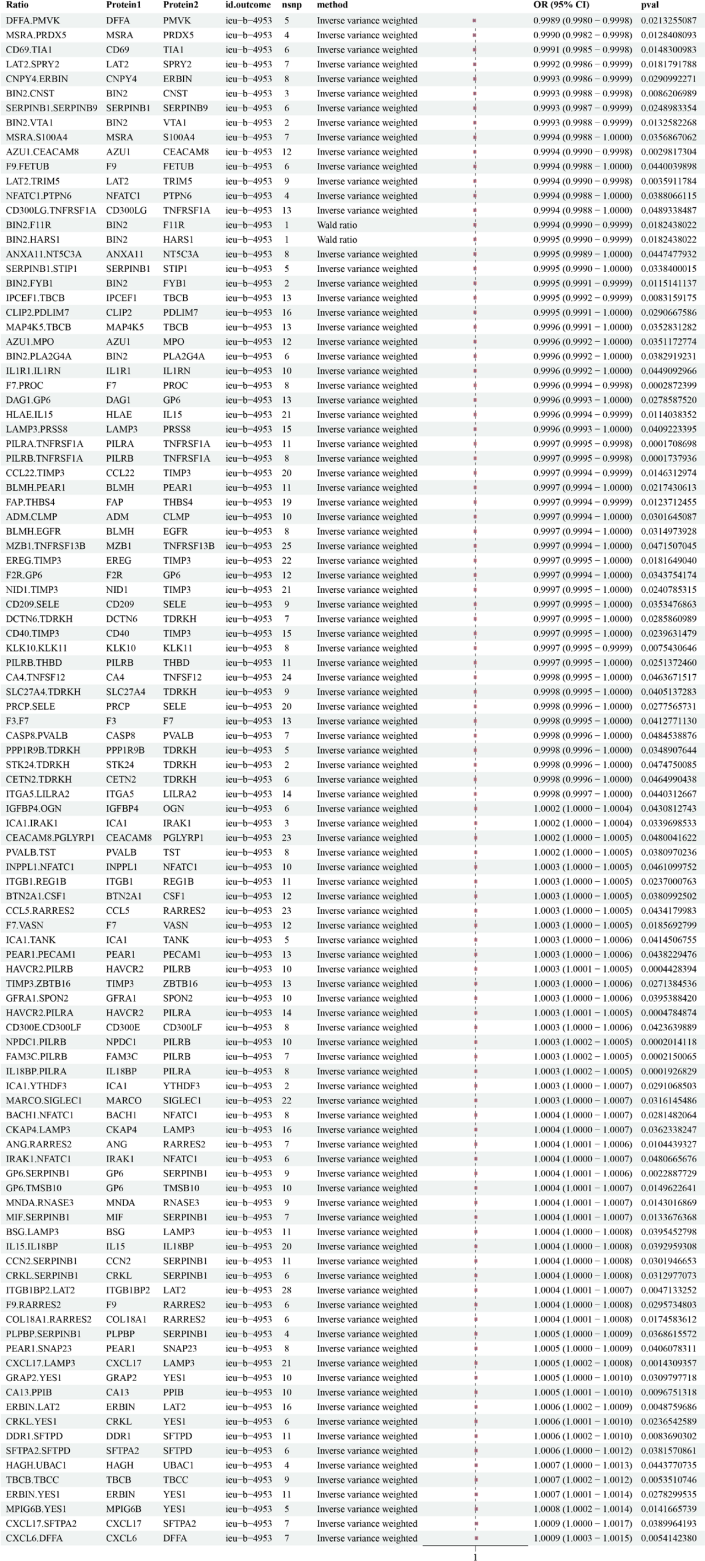
Primary analysis of causal associations between protein–protein ratio and HCC pathogenesis using the IEU database.

In the IEU OpenGWAS database, 34,268 SNPs associated with HCC were screened for MR analysis after excluding confounders such as hepatitis, liver cirrhosis, fatty liver, and alcohol consumption (Table S1). The screening results are detailed in Data S3. The main results of the analysis of the causal association between the protein‐to‐protein ratio and HCC morbidity risk are shown in Figure 3. The results showed that LAT2/SPRY2 (OR: 0.9992, 95% CI: 0.9986–0.9999; *p* = 0.018), FAP/THBS4 (OR: 0.9997, 95% CI: 0.9994–0.9999; *p* = 0.012), ICA1/IRAK1 (OR: 1.0002, 95% CI: 1–1.0004; *p* = 0.033), ERBIN/LAT2 (OR: 1.0006, 95% CI: 1.0002–1.0009); and ITGB1BP2/LAT2 (OR: 1.0004, 95% CI: 1.0001–1.0007; *p* < 0.005), a total of 51 protein pairs, were linked to an increased risk of HCC. The results of the heterogeneity and horizontal pleiotropy analyses of rQTL associated with HCC (ieu‐b‐4953) are shown in Data S4.

We considered the intersection of positive results from the two databases. A total of eight protein–protein pairs were causally associated with the risk of HCC morbidity in the FinnGen and IEU OpenGWAS databases (Table 2). The effects of ERBIN/LAT2, ICA1/IRAK1, ICA1/YTHDF3, ITGB1BP2/LAT2, LAT2/SPRY2, and FAP/THBS4 showed consistent directionality across the FinnGen and IEU databases.

**TABLE 2:** Intersection of the positive results in the FinnGen and IEU databases.

Ratio	Protein1	Protein2	Outcome	Nsnp	Methods	OR (95% CI)	*p*
ERBIN.LAT2	ERBIN	LAT2	finngen_R11_HCC	20	Inverse variance weighted	1.9763 (1.3009–3.0026)	0.00141
ERBIN.LAT2	ERBIN	LAT2	ieu‐b‐4953	16	Inverse variance weighted	1.0006 (1.0002–1.0009)	0.004876
FAP.THBS4	FAP	THBS4	finngen_R11_HCC	21	Inverse variance weighted	0.6537 (0.4919–0.8687)	0.003384
FAP.THBS4	FAP	THBS4	ieu‐b‐4953	19	Inverse variance weighted	0.9997 (0.9994–0.9999)	0.012371
ICA1.IRAK1	ICA1	IRAK1	finngen_R11_HCC	5	Inverse variance weighted	1.3014 (1.0345–1.637)	0.024458
ICA1.IRAK1	ICA1	IRAK1	ieu‐b‐4953	3	Inverse variance weighted	1.0002 (1–1.0004)	0.03397
ICA1.YTHDF3	ICA1	YTHDF3	finngen_R11_HCC	2	Inverse variance weighted	1.4362 (1.0052–2.0518)	0.046741
ICA1.YTHDF3	ICA1	YTHDF3	ieu‐b‐4953	2	Inverse variance weighted	1.0003 (1–1.0007)	0.029107
ITGB1BP2.LAT2	ITGB1BP2	LAT2	finngen_R11_HCC	33	Inverse variance weighted	1.5055 (1.0418–2.1755)	0.029411
ITGB1BP2.LAT2	ITGB1BP2	LAT2	ieu‐b‐4953	28	Inverse variance weighted	1.0004 (1.0001–1.0007)	0.004713
LAT2.SPRY2	LAT2	SPRY2	finngen_R11_HCC	11	Inverse variance weighted	0.4974 (0.2506–0.9871)	0.045805
LAT2.SPRY2	LAT2	SPRY2	ieu‐b‐4953	7	Inverse variance weighted	0.9992 (0.9986–0.9999)	0.018179
PPP1R9B.TDRKH	PPP1R9B	TDRKH	finngen_R11_HCC	8	Inverse variance weighted	1.265 (1.0043–1.5933)	0.045856
PPP1R9B.TDRKH	PPP1R9B	TDRKH	ieu‐b‐4953	5	Inverse variance weighted	0.9998 (0.9996–1)	0.034891
STK24.TDRKH	STK24	TDRKH	finngen_R11_HCC	4	Inverse variance weighted	1.2839 (1.0031–1.6434)	0.047222
STK24.TDRKH	STK24	TDRKH	ieu‐b‐4953	2	Inverse variance weighted	0.9998 (0.9996–1)	0.047475

### 3.2 Steiger Direction Test

The results showed that the direction of the eight positive protein–protein pairs and the HCC outcome was “TRUE,” indicating that the causal relationship between the above protein‐to‐protein ratio and HCC was consistent with the expected direction (Table 3).

**TABLE 3:** Steiger direction test results.

Outcome	Exposure	Correct_causal_direction	*p*
Hepatocellular carcinoma (finngen‐R11‐HCC)	ERBIN/LAT2	TRUE	< 0.001
Hepatocellular carcinoma (finngen‐R11‐HCC)	FAP/THBS4	TRUE	< 0.001
Hepatocellular carcinoma (finngen‐R11‐HCC)	ICA1/IRAK1	TRUE	< 0.001
Hepatocellular carcinoma (finngen‐R11‐HCC)	ICA1/YTHDF3	TRUE	< 0.001
Hepatocellular carcinoma (finngen‐R11‐HCC)	ITGB1BP2/LAT2	TRUE	< 0.001
Hepatocellular carcinoma (finngen‐R11‐HCC)	LAT2/SPRY2	TRUE	< 0.001
Hepatocellular carcinoma (finngen‐R11‐HCC)	PPP1R9B/TDRKH	TRUE	< 0.001
Hepatocellular carcinoma (finngen‐R11‐HCC)	STK24/TDRKH	TRUE	< 0.001
Liver cell carcinoma (ieu‐b‐4953)	ERBIN/LAT2	TRUE	< 0.001
Liver cell carcinoma (ieu‐b‐4953)	FAP/THBS4	TRUE	< 0.001
Liver cell carcinoma (ieu‐b‐4953)	ICA1/IRAK1	TRUE	< 0.001
Liver cell carcinoma (ieu‐b‐4953)	ICA1/YTHDF3	TRUE	< 0.001
Liver cell carcinoma (ieu‐b‐4953)	ITGB1BP2/LAT2	TRUE	< 0.001
Liver cell carcinoma (ieu‐b‐4953)	LAT2/SPRY2	TRUE	< 0.001
Liver cell carcinoma (ieu‐b‐4953)	PPP1R9B/TDRKH	TRUE	< 0.001
Liver cell carcinoma (ieu‐b‐4953)	STK24/TDRKH	TRUE	< 0.001

### 3.3 Co‐Localization Analysis

The results of the co‐localization analysis revealed weak evidence of co‐localization of positive protein–protein pairs with HCC (Table S2).

### 3.4 Causal Association Between Circulating Proteins and HCC

For the eight positive protein pairs, the causal effect of a single circulating protein on the risk of HCC was explored based on two‐sample MR, drug–target MR, and co‐localization analysis.

### 3.5 Two‐Sample MR

**FIGURE 4:**
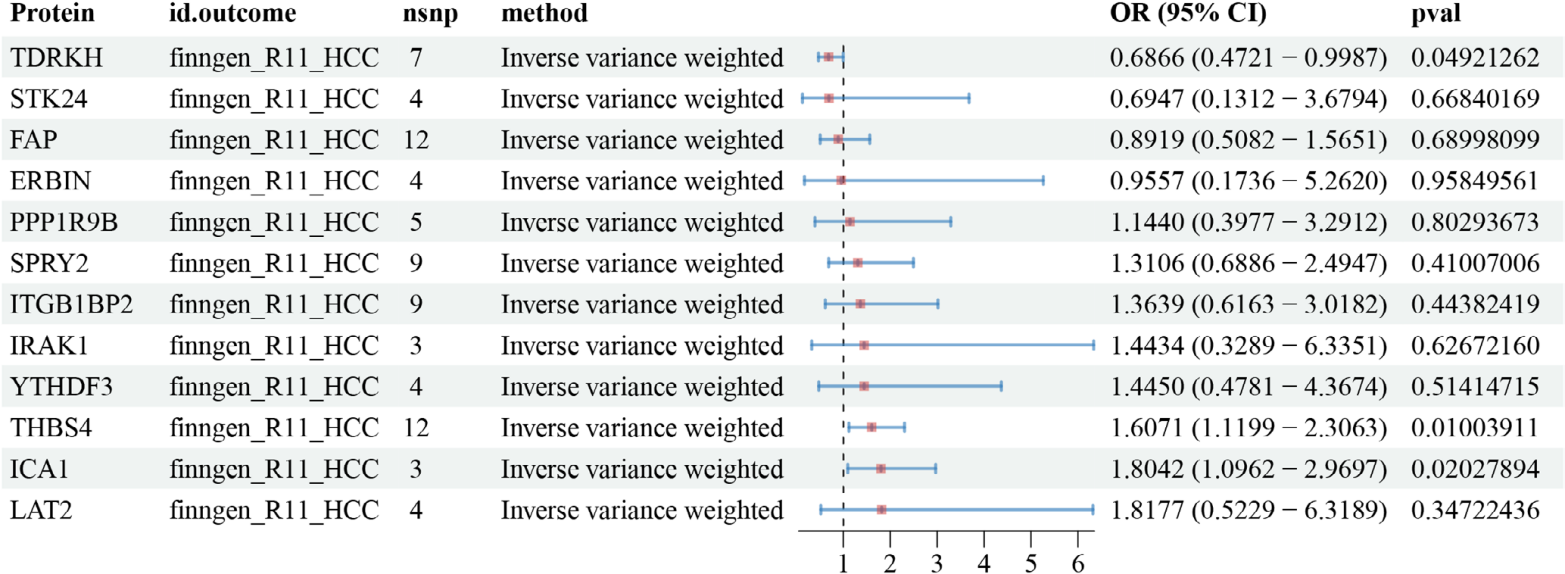
TSMR results of individual proteins and HCC. TSMR, two‐sample Mendelian randomization.

The results of the two‐sample MR analysis showed that three proteins, TDRKH (OR: 0.6866, 95% CI: 0.4721–0.9987; *p* = 0.049), THBS4 (OR: 1.6071, 95% CI: 1.1199–2.3063; *p* = 0.01), and ICA1 (OR: 1.8042, 95% CI: 1.0962–2.9697; *p* = 0.02), were causally associated with HCC (Figure 4). Sensitivity analysis results for pQTLs associated with HCC (finngen‐R11‐HCC) are detailed in Table S3.

### 3.6 Co‐Localization Analysis of Common Two‐Sample MR

The results of the co‐localization analysis suggested weak evidence of co‐localization between pQTLs in positive protein pairs and HCC (Table S4).

### 3.7 Drug‐Target MR

**FIGURE 5:**
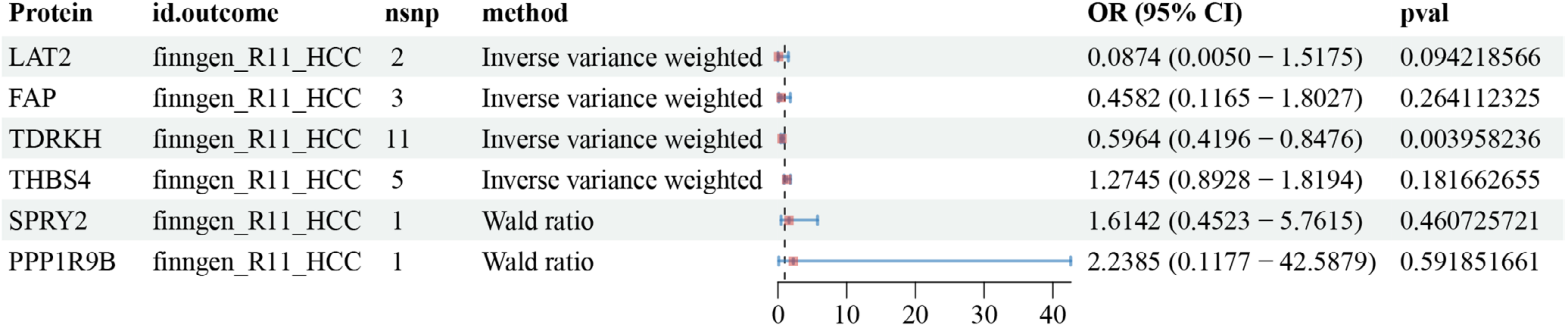
MR results of single protein and drug‐target in HCC. MR, Mendelian randomization.

The results of the drug target MR showed that only the TDRKH (OR: 0.5964, 95% CI: 0.4196–0.8476; *p* < 0.005) protein was causally associated with HCC (Figure 5). The results of the sensitivity analysis of the pQTLs associated with HCC (finngen‐R11‐HCC) are shown in Table S3.

## 4 Discussion

The mechanism of HCC morbidity is complex and involves cell cycle deregulation, chromosomal instability, immune regulation, epithelial‐mesenchymal transition, and microRNA deregulation [33]. The combination therapy with ICI has significantly improved the treatment outcomes for advanced HCC. This therapeutic approach is not only used for the treatment of unresectable liver cancer but also serves as a bridge and downstaging treatment before liver transplantation, as well as a reference for postoperative tumor recurrence management [34, 35]. The main challenges regarding HCC are early and accurate diagnosis and medical therapy after diagnosis [36, 37]. Therefore, there is an urgent need to identify new biomarkers and therapeutic targets for the treatment of HCC. To our knowledge, this study is the first to assess the association between 2821 pairs of plasma protein‐to‐protein ratios (rQTLs) and the risk of HCC morbidity. After screening and intersecting samples from two different populations, we identified eight plasma protein–protein pairs (ERBIN/LAT2, ICA1/IRAK1, ICA1/YTHDF3, ITGB1BP2/LAT2, LAT2/SPRY2, FAP/THBS4, PPP1R9B/TDRKH, and STK24/TDRKH) that were strongly associated with HCC. From the above eight positive protein–protein pairs, MR analysis of pure plasma circulating proteins (pQTL) showed that one protein (TDRKH) was robustly and significantly associated with the pathogenesis of HCC.

Interleukin receptor‐associated kinase 1 (IRAK1) reportedly plays an important role in a series of malignant tumors and is overexpressed in HCC tissues and cell lines. RAK1/4 inhibitors cause G1/S cell cycle arrest and apoptosis, confirming that IRAK1 is a new target for drug therapy for HCC [38]. THBS4 is part of the extracellular matrix, and its regulation of HCC progression is related to the interaction of Integrin β1, which involves the FAK/PI3K/AKT signaling pathway [39] and may be a prognostic marker or potential therapeutic target for HCC [40]. SPRY2 expression is downregulated in various cancers, including prostate, liver, lung, and breast cancers [41]. One study found that the inhibition of SPRY2 expression via the MAPK/ERK signaling pathway promotes HCC development [42]. It has been reported that activating the STAT3 pathway promotes the expression of fibroblast activation protein (FAP) [43], thereby mediating proliferation and migration of HCC cells while inhibiting apoptosis of HCC cells. FAP can be used as a marker of poor prognosis in patients with HCC [44, 45]. ERBIN promotes HCC by inactivating ERα‐mediated tumor suppressor signaling [46]. The roles of these molecules in HCC are consistent with our results. Additionally, we found a strong causal association between other circulating proteins and HCC risk. Among them, high expression of L‐type amino acid transporter 2 (LAT2) leads to abnormal amino acid uptake and metabolism, causing rapid proliferation in pancreatic cancer [47], osteosarcoma [48], and other tumor cells. Small‐molecule inhibitors of LAT2 reportedly inhibit CD47‐mediated tumor immune escape [49]. YTHDF3 is a reader of N6‐methyladenosine (m^6^A), and recent studies have revealed that YTHDF3 plays a role in tumorigenesis by recognizing the m^6^A modification in MYC mRNA. This highlights the therapeutic potential of targeting the YTHDF3‐MYC signaling axis in pancreatic cancer [50]. Results from a proximity extension assay study suggested that ITGB1BP2 may be a candidate biomarker for diagnosing invasive cervical cancer [51]. PPP1R9B (SPN or NEURABIN‐2) is a tumor suppressor that affects tumor initiation and progression [52, 53] when the SPN domain that interacts with protein phosphokinase 1 (PP1) is mutated. Recent studies have found that the expression of the serine–threonine kinase (STK24), which regulates tumor immune escape by promoting AKT phosphorylation and PD‐L1 expression, is upregulated in tumor specimens [54]. The role of ICA1 in gliomas requires further study [55]. In light of the above mechanisms in other cancers, the specific mechanisms underlying the roles of these circulating proteins in HCC development are yet to be further verified and explored.

MR analysis of single circulating proteins based on the eight positive protein–protein pairs yielded one protein (TDRKH). Few reports exist on TDRKH; however, some studies have shown that TDRKH‐AS1 (an antisense RNA of TDRKH) is highly expressed in HCC cell lines in vitro. TDRKH‐AS1 knockdown may affect cell proliferation by inducing apoptosis in HCC cells [56]. Additionally, TDRKH‐AS1 is upregulated in patients with colorectal cancer and can target β‐catenin in the Wnt signaling pathway to exert oncogenic activity [57]. TDRKH‐AS1 can be used as a prognostic marker for HCC [56], which is consistent with our findings. Similar to a previous paper on cell genomics [21], only one protein (TDRKH) was found to have a positive causal association with HCC based on a single pQTL in this study, while eight robust protein–protein pairs were found to have a causal association with HCC after considering protein–protein interactions. Therefore, considering protein–protein interactions will help identify new targets for diagnosis and intervention.

This study has several significant strengths. First, genetic data on the plasma protein ratio and HCC were derived from numerous GWAS designed to minimize sample overlap, thereby reducing the risk of confounding effects due to shared samples between datasets. Second, the inclusion of different HCC cohorts in the FinnGen and UKB datasets helped mitigate potential confounding effects due to population differences. Third, the F‐statistics of all selected genetic instrumental variables exceeded 10, indicating that the instrumental variable intensity was high. This could effectively reduce the bias introduced by weak instrumental variables, thus improving the credibility of the instrumental variables used in this analysis. Finally, integrating multiple sensitivity analysis techniques enhanced the reliability of the results.

Although the analysis in this study was relatively comprehensive, several limitations must be acknowledged that may affect our interpretation of the results. First, the limitations inherent in MR must be recognized, including issues such as heterogeneity in phenotypic traits and developmental compensation, which may have undermined the accuracy and applicability of our findings [58]. Second, we relied only on aggregate‐level data, which limited our ability to perform hierarchical analysis or fully explore individual‐level data. Future studies could conduct more in‐depth analyses based on individual‐level data and perform stratified analyses for HCC caused by different etiologies. Third, given that the study population was composed primarily of individuals of European ancestry, it is prudent to generalize the results to more heterogeneous groups, such as Asians. Follow‐up studies should be conducted in different ethnic groups to verify the relevance of our findings. Fourth, it is important to note that no correction for multiple testing was performed in this study; however, we used two large‐sample datasets to maximize the robustness of the results through sensitivity analysis. Finally, although we revealed a causal relationship between the eight protein pairs and HCC, our understanding of the underlying mechanisms is incomplete. Further studies are needed to elucidate these complex pathways.

In conclusion, based on the rQTL and pQTL data, we found a causal relationship between HCC and eight protein–protein pairs and one protein and HCC, providing new insights into the diagnosis and treatment of HCC.

## Author Contributions

Xu Shaohua, Zhang Mingwei, and Li Yueming supervised the entire project and designed the work. Yue Qiuyuan contributed to the data analysis, data interpretation, manuscript writing, and revision. Li Xiaoxia contributed to the data analysis. Wan Xiaoye contributed to manuscript writing. Lin Xi contributed to data curation. All authors reviewed or revised the manuscript and approved the final draft for submission.

## Ethics Statement

This study was performed in line with the principles of the Declaration of Helsinki.

## Consent

Written informed consent for participation was not required for this study in accordance with national legislation and institutional requirements. No animal studies are presented in this manuscript.

## Conflicts of Interest

The authors declare no conflicts of interest.

## Supporting information


**Data S1** Details of SNPs for Mendelian randomization analysis based on the FinnGen database.

**Data S2** Heterogeneity and pleiotropy analyses of rQTLs associated with hepatocellular carcinoma (HCC) (finngen‐R11‐HCC).

**Data S3** Details of SNPs for Mendelian randomization analysis based on the IEU OpenGWAS database.

**Data S4** Heterogeneity and pleiotropy analyses of rQTLs associated with HCC (ieu‐b‐4953).

**Table S1** Excluded confounding factors.

**Table S2** Co‐localization analyses of positive protein–protein pairs with HCC.

**Table S3** Sensitivity analyses of protein quantitative trait loci (pQTLs) associated with HCC (finngen‐R11‐HCC).

**Table S4** Co‐localization analyses of positive proteins with HCC.

## Data Availability

The summary statistics for the protein–protein ratios are available in the research of Karsten Suhre (https://doi.org:10.1016/j.xgen.2024.100506). The HCC summary statistics for the FinnGen GWAS are available at https://www.finngen.fi/en/access_results. The HCC summary statistics for the IEU OpenGWAS project are available at https://gwas.mrcieu.ac.uk/datasets/.

## References

[1] K. A. McGlynn , J. L. Petrick , and H. B. El‐Serag , “Epidemiology of Hepatocellular Carcinoma,” Hepatology 73, no. Suppl 1 (2021): 4–13, 10.1002/hep.31288. PMC7577946 32319693

[2] M. Tariq , A. B. Shoukat , S. Akbar , et al., “Epidemiology, Risk Factors, and Pathogenesis Associated With a Superbug: A Comprehensive Literature Review on Hepatitis C Virus Infection,” Sage Open Medicine 10 (2022): 20503121221105957, 10.1177/20503121221105957. 35795865 PMC9252020

[3] A. Perisetti , H. Goyal , R. Yendala , R. B. Thandassery , and E. Giorgakis , “Non‐Cirrhotic Hepatocellular Carcinoma in Chronic Viral Hepatitis: Current Insights and Advancements,” World Journal of Gastroenterology 27 (2021): 3466–3482, 10.3748/wjg.v27.i24.3466. 34239263 PMC8240056

[4] G. Sapisochin and J. Bruix , “Liver Transplantation for Hepatocellular Carcinoma: Outcomes and Novel Surgical Approaches,” Nature Reviews. Gastroenterology & Hepatology 14 (2017): 203–217, 10.1038/nrgastro.2016.193. 28053342

[5] C. J. Wehrle , R. Raj , M. Maspero , et al., “Risk Assessment in Liver Transplantation for Hepatocellular Carcinoma: Long‐Term Follow‐Up of a Two‐Centre Experience,” International Journal of Surgery 110 (2024): 2818–2831, 10.1097/JS9.0000000000001104. 38241354 PMC11093438

[6] C. J. Wehrle , J. Kusakabe , M. Akabane , et al., “Expanding Selection Criteria in Deceased Donor Liver Transplantation for Hepatocellular Carcinoma: Long‐Term Follow‐Up of a National Registry and 2 Transplant Centers,” Transplantation 108 (2024): 2386–2395, 10.1097/TP.0000000000005097. 38831488

[7] M. Guo , H. Zhang , J. Zheng , and Y. Liu , “Glypican‐3: A New Target for Diagnosis and Treatment of Hepatocellular Carcinoma,” Journal of Cancer 11 (2020): 2008–2021, 10.7150/jca.39972. 32127929 PMC7052944

[8] S. Yang , L. Pang , W. Dai , et al., “Role of Forkhead Box O Proteins in Hepatocellular Carcinoma Biology and Progression (Review),” Frontiers in Oncology 11 (2021): 667730, 10.3389/fonc.2021.667730. 34123834 PMC8190381

[9] S. Pathak and M. B. Sonbol , “Second‐Line Treatment Options for Hepatocellular Carcinoma: Current Landscape and Future Direction,” Journal of Hepatocellular Carcinoma 8 (2021): 1147–1158, 10.2147/JHC.S268314. 34584898 PMC8464222

[10] Q. Xie , P. Zhang , Y. Wang , W. Mei , and C. Zeng , “Overcoming Resistance to Immune Checkpoint Inhibitors in Hepatocellular Carcinoma: Challenges and Opportunities,” Frontiers in Oncology 12 (2022): 958720, 10.3389/fonc.2022.958720. 36119533 PMC9478417

[11] Z. Cai , X. Su , L. Qiu , et al., “Personalized Neoantigen Vaccine Prevents Postoperative Recurrence in Hepatocellular Carcinoma Patients With Vascular Invasion,” Molecular Cancer 20 (2021): 164, 10.1186/s12943-021-01467-8. 34903219 PMC8667400

[12] A. Kopystecka , R. Patryn , M. Lesniewska , J. Budzynska , and I. Koziol , “The Use of ctDNA in the Diagnosis and Monitoring of Hepatocellular Carcinoma‐Literature Review,” International Journal of Molecular Sciences 24 (2023): 9342, 10.3390/ijms24119342. 37298294 PMC10253340

[13] C. J. Wehrle , H. Hong , S. Kamath , et al., “Tumor Mutational Burden From Circulating Tumor DNA Predicts Recurrence of Hepatocellular Carcinoma After Resection: An Emerging Biomarker for Surveillance,” Annals of Surgery 280 (2024): 504–513, 10.1097/SLA.0000000000006386. 38860385

[14] J. von Felden , A. J. Craig , T. Garcia‐Lezana , et al., “Mutations in Circulating Tumor DNA Predict Primary Resistance to Systemic Therapies in Advanced Hepatocellular Carcinoma,” Oncogene 40 (2021): 140–151, 10.1038/s41388-020-01519-1. 33097857 PMC12452111

[15] R. Raj , C. J. Wehrle , N. Aykun , et al., “Immunotherapy Plus Locoregional Therapy Leading to Curative‐Intent Hepatectomy in HCC: Proof of Concept Producing Durable Survival Benefits Detectable With Liquid Biopsy,” Cancers (Basel) 15 (2023): 5220, 10.3390/cancers15215220. 37958394 PMC10650763

[16] X. Bessa , J. Vidal , J. C. Balboa , et al., “High Accuracy of a Blood ctDNA‐Based Multimodal Test to Detect Colorectal Cancer,” Annals of Oncology 34 (2023): 1187–1193, 10.1016/j.annonc.2023.09.3113. 37805131

[17] N. Suna , D. Ozer Etik , S. Ocal , and H. Selcuk , “Effect of Propranolol Treatment on the Incidence of Hepatocellular Carcinoma in Patients Waiting for Liver Transplant With Cirrhosis: A Retrospective, Surveillance Study in a Tertiary Center,” Experimental and Clinical Transplantation 17 (2019): 632–637, 10.6002/ect.2018.0321. 31050621

[18] J. Liu , K. Park , Z. Shen , et al., “Immunotherapy, Targeted Therapy, and Their Cross Talks in Hepatocellular Carcinoma,” Frontiers in Immunology 14 (2023): 1285370, 10.3389/fimmu.2023.1285370. 38173713 PMC10762788

[19] J. Zheng , D. Baird , M. C. Borges , et al., “Recent Developments in Mendelian Randomization Studies,” Current Epidemiology Reports 4 (2017): 330–345, 10.1007/s40471-017-0128-6. 29226067 PMC5711966

[20] S. Burgess , D. S. Small , and S. G. Thompson , “A Review of Instrumental Variable Estimators for Mendelian Randomization,” Statistical Methods in Medical Research 26 (2017): 2333–2355, 10.1177/0962280215597579. 26282889 PMC5642006

[21] K. Suhre , “Genetic Associations With Ratios Between Protein Levels Detect New pQTLs and Reveal Protein‐Protein Interactions,” Cell Genomics 4 (2024): 100506, 10.1016/j.xgen.2024.100506. 38412862 PMC10943581

[22] B. B. Sun , J. Chiou , M. Traylor , et al., “Genetic Regulation of the Human Plasma Proteome in 54,306 UK Biobank Participants,” (2022). *bioRxiv*, 10.1101/2022.06.17.496443.

[23] M. I. Kurki , J. Karjalainen , P. Palta , et al., “FinnGen Provides Genetic Insights From a Well‐Phenotyped Isolated Population,” Nature 613 (2023): 508–518, 10.1038/s41586-022-05473-8. 36653562 PMC9849126

[24] C. J. B. Kimberley Burrows , T. Dudding , M. Gormley , et al., “Genome‐Wide Association Study of Cancer Risk in UK Biobank” (2021), University of Bristol, 10.5523/bris.aed0u12w0ede20olb0m77p4b9.

[25] G. Hemani , J. Zheng , B. Elsworth , et al., “The MR‐Base Platform Supports Systematic Causal Inference Across the Human Phenome,” eLife 7 (2018): e34408, 10.7554/eLife.34408. 29846171 PMC5976434

[26] V. Zuber , N. F. Grinberg , D. Gill , et al., “Combining Evidence From Mendelian Randomization and Colocalization: Review and Comparison of Approaches,” American Journal of Human Genetics 109 (2022): 767–782, 10.1016/j.ajhg.2022.04.001. 35452592 PMC7612737

[27] M. Arnold , J. Raffler , A. Pfeufer , K. Suhre , and G. Kastenmuller , “SNiPA: An Interactive, Genetic Variant‐Centered Annotation Browser,” Bioinformatics 31 (2015): 1334–1336, 10.1093/bioinformatics/btu779. 25431330 PMC4393511

[28] S. Burgess , S. G. Thompson , and Collaboration, C. C. G , “Avoiding Bias From Weak Instruments in Mendelian Randomization Studies,” International Journal of Epidemiology 40 (2011): 755–764, 10.1093/ije/dyr036. 21414999

[29] C. Giambartolomei , D. Vukcevic , E. E. Schadt , et al., “Bayesian Test for Colocalisation Between Pairs of Genetic Association Studies Using Summary Statistics,” PLoS Genetics 10 (2014): e1004383, 10.1371/journal.pgen.1004383. 24830394 PMC4022491

[30] J. Chen , F. Xu , X. Ruan , et al., “Therapeutic Targets for Inflammatory Bowel Disease: Proteome‐Wide Mendelian Randomization and Colocalization Analyses,” eBioMedicine 89 (2023): 104494, 10.1016/j.ebiom.2023.104494. 36857861 PMC9986512

[31] S. M. Lutz , K. Voorhies , A. C. Wu , J. Hokanson , S. Vansteelandt , and C. Lange , “The Influence of Unmeasured Confounding on the MR Steiger Approach,” Genetic Epidemiology 46 (2022): 139–141, 10.1002/gepi.22442. 35170805 PMC8915443

[32] J. Bowden , G. Davey Smith , and S. Burgess , “Mendelian Randomization With Invalid Instruments: Effect Estimation and Bias Detection Through Egger Regression,” International Journal of Epidemiology 44 (2015): 512–525, 10.1093/ije/dyv080. 26050253 PMC4469799

[33] H. Shinoda , “Effect of Long‐Term Administration of Fluoride on Physico‐Chemical Properties of the Rat Incisor Enamel,” Calcified Tissue Research 18 (1975): 91–100, 10.1007/BF02546229. 1148900

[34] P. Tabrizian , M. L. Holzner , V. Ajmera , et al., “Intention‐To‐Treat Outcomes of Patients With Hepatocellular Carcinoma Receiving Immunotherapy Before Liver Transplant: The Multicenter VITALITY Study,” Journal of Hepatology (2024), 10.1016/j.jhep.2024.09.003. 39255928

[35] M. S. Rezaee‐Zavareh , Y. H. Yeo , T. Wang , et al., “Impact of Pre‐Transplant Immune Checkpoint Inhibitor Use on Post‐Transplant Outcomes in HCC: A Systematic Review and Individual Patient Data Meta‐Analysis,” Journal of Hepatology 82 (2024): 107–119, 10.1016/j.jhep.2024.06.042. 38996924 PMC11655254

[36] Z. Wang , H. Qin , S. Liu , J. Sheng , and X. Zhang , “Precision Diagnosis of Hepatocellular Carcinoma,” Chinese Medical Journal 136 (2023): 1155–1165, 10.1097/CM9.0000000000002641. 36939276 PMC10278703

[37] J. D. Yang , P. Hainaut , G. J. Gores , A. Amadou , A. Plymoth , and L. R. Roberts , “A Global View of Hepatocellular Carcinoma: Trends, Risk, Prevention and Management,” Nature Reviews. Gastroenterology & Hepatology 16 (2019): 589–604, 10.1038/s41575-019-0186-y. 31439937 PMC6813818

[38] N. Li , J. Jiang , J. Fu , et al., “Targeting Interleukin‐1 Receptor‐Associated Kinase 1 for Human Hepatocellular Carcinoma,” Journal of Experimental & Clinical Cancer Research 35 (2016): 140, 10.1186/s13046-016-0413-0. 27619757 PMC5020546

[39] D. G. Hughes , E. A. Dowling , R. E. DeMeersman , W. R. Garnett , and H. T. Karnes , “Cardiovascular Effects of H2‐Receptor Antagonists,” Journal of Clinical Pharmacology 29 (1989): 472–477, 10.1002/j.1552-4604.1989.tb03365.x. 2567740

[40] H. Wu , G. Zhang , Z. Li , et al., “Thrombospondin‐4 Expression as a Prognostic Marker in Hepatocellular Carcinoma,” Gene 696 (2019): 219–224, 10.1016/j.gene.2019.02.049. 30802535

[41] H. Dai , W. Xu , L. Wang , et al., “Loss of SPRY2 Contributes to Cancer‐Associated Fibroblasts Activation and Promotes Breast Cancer Development,” Breast Cancer Research 25 (2023): 90, 10.1186/s13058-023-01683-8. 37507768 PMC10375677

[42] S. Xiao , M. Yang , H. Yang , R. Chang , F. Fang , and L. Yang , “miR‐330‐5p Targets SPRY2 to Promote Hepatocellular Carcinoma Progression via MAPK/ERK Signaling,” Oncogenesis 7 (2018): 90, 10.1038/s41389-018-0097-8. 30464168 PMC6249243

[43] D. Sun , W. Li , D. Ding , et al., “IL‐17a Promotes Hepatocellular Carcinoma by Increasing FAP Expression in Hepatic Stellate Cells via Activation of the STAT3 Signaling Pathway,” Cell Death Discovery 10 (2024): 230, 10.1038/s41420-024-01995-4. 38740736 PMC11091202

[44] H. Y. Woo , H. Rhee , J. E. Yoo , et al., “Lung and Lymph Node Metastases From Hepatocellular Carcinoma: Comparison of Pathological Aspects,” Liver International 42 (2022): 199–209, 10.1111/liv.15051. 34490997

[45] X. Wang , R. Niu , H. Yang , Y. Lin , H. Hou , and H. Yang , “Fibroblast Activation Protein Promotes Progression of Hepatocellular Carcinoma via Regulating the Immunity,” Cell Biology International 48 (2024): 577–593, 10.1002/cbin.12154. 38501437

[46] H. Wu , S. Yao , S. Zhang , et al., “Elevated Expression of Erbin Destabilizes ERalpha Protein and Promotes Tumorigenesis in Hepatocellular Carcinoma,” Journal of Hepatology 66 (2017): 1193–1204, 10.1016/j.jhep.2017.01.030. 28192186

[47] M. Feng , G. Xiong , Z. Cao , et al., “LAT2 Regulates Glutamine‐Dependent mTOR Activation to Promote Glycolysis and Chemoresistance in Pancreatic Cancer,” Journal of Experimental & Clinical Cancer Research 37 (2018): 274, 10.1186/s13046-018-0947-4. 30419950 PMC6233565

[48] E. G. E. Hurkmans , J. B. Koenderink , J. J. M. W. van den Heuvel , et al., “SLC7A8 Coding for LAT2 Is Associated With Early Disease Progression in Osteosarcoma and Transports Doxorubicin,” Frontiers in Pharmacology 13 (2022): 1042989, 10.3389/fphar.2022.1042989. 36438828 PMC9681801

[49] Z. Wang , B. Li , S. Li , et al., “Metabolic Control of CD47 Expression Through LAT2‐Mediated Amino Acid Uptake Promotes Tumor Immune Evasion,” Nature Communications 13 (2022): 6308, 10.1038/s41467-022-34064-4. PMC9588779 36274066

[50] H. Zhang , Y. Sun , Z. Wang , et al., “ZDHHC20‐Mediated S‐Palmitoylation of YTHDF3 Stabilizes MYC mRNA to Promote Pancreatic Cancer Progression,” Nature Communications 15 (2024): 4642, 10.1038/s41467-024-49105-3. PMC11143236 38821916

[51] M. Berggrund , S. Enroth , M. Lundberg , et al., “Identification of Candidate Plasma Protein Biomarkers for Cervical Cancer Using the Multiplex Proximity Extension Assay,” Molecular & Cellular Proteomics 18 (2019): 735–743, 10.1074/mcp.RA118.001208. 30692274 PMC6442356

[52] E. M. Verdugo‐Sivianes and A. Carnero , “SPINOPHILIN: A Multiplayer Tumor Suppressor,” Genes & Diseases 10 (2023): 187–198, 10.1016/j.gendis.2021.12.021. 37013033 PMC10066247

[53] E. M. Verdugo‐Sivianes , A. M. Rojas , S. Munoz‐Galvan , D. Otero‐Albiol , and A. Carnero , “Mutation of SPINOPHILIN (PPP1R9B) Found in Human Tumors Promotes the Tumorigenic and Stemness Properties of Cells,” Theranostics 11 (2021): 3452–3471, 10.7150/thno.53572. 33537097 PMC7847670

[54] N. Wang , Y. Jiang , M. Li , et al., “Protein Kinase STK24 Promotes Tumor Immune Evasion via the AKT‐PD‐L1 Axis,” Advanced Science 11 (2024): e2304342, 10.1002/advs.202304342. 38229183 PMC10966517

[55] Y. Jin , Z. Wang , K. Xiang , et al., “Comprehensive Development and Validation of Gene Signature for Predicting Survival in Patients With Glioblastoma,” Frontiers in Genetics 13 (2022): 900911, 10.3389/fgene.2022.900911. 36035145 PMC9399759

[56] X. Bu , L. Ma , S. Liu , et al., “A Novel Qualitative Signature Based on lncRNA Pairs for Prognosis Prediction in Hepatocellular Carcinoma,” Cancer Cell International 22 (2022): 95, 10.1186/s12935-022-02507-z. 35193591 PMC8862507

[57] Y. Jiao , J. Zhou , Y. Jin , et al., “Long Non‐Coding RNA TDRKH‐AS1 Promotes Colorectal Cancer Cell Proliferation and Invasion Through the Beta‐Catenin Activated Wnt Signaling Pathway,” Frontiers in Oncology 10 (2020): 639, 10.3389/fonc.2020.00639. 32670860 PMC7326065

[58] P. C. Haycock , S. Burgess , K. H. Wade , J. Bowden , C. Relton , and G. Davey Smith , “Best (But Oft‐Forgotten) Practices: The Design, Analysis, and Interpretation of Mendelian Randomization Studies,” American Journal of Clinical Nutrition 103 (2016): 965–978, 10.3945/ajcn.115.118216. 26961927 PMC4807699

